# Spad value varies with age and leaf of maize plant and its relationship with grain yield

**DOI:** 10.1186/s13104-020-05324-7

**Published:** 2020-10-08

**Authors:** Bishnu Prasad Kandel

**Affiliations:** Department of Plant Breeding, Post Graduate Program, Institute of Agriculture and Animals Science, Tribhuvan University, Kirtipur, Nepal

**Keywords:** Corn, Chlorophyll concentration, N concentration, SPAD etc

## Abstract

**Objectives:**

A field experiment was conducted to evaluate Soil Plant Analysis Development (SPAD) value in different age and leaf of maize hybrid and correlating with grain yield. Ten maize hybrids were replicated thricely under Randomized Complete Block Design (RCBD) during winter of 2018. SPAD value was measured by SPAD 502 plus meter. At 30 days interval during vegetative stage SPAD measurement were taken from T1 (top most leaf) and T3 (2nd leaf from top leaf) leaves of five randomly selected plants from one plot and they were averaged. For reproductive phase data taken from eo (leaf attached to ear) and e2 (2nd leaf from eo leaf) leaves at 10 days intervals. Same leaves were used for entire data collection.

**Results:**

Significantly different SPAD value was observed for different age and leaves of maize during pre and post anthesis. SPAD value increase with increase in age and decrease at the time of maturity. During vegetative phase T3 leaves has more SPAD value than T1. During reproductive stage eo leaves had more SPAD than e2 leaves, so center leaf of maize contributes more to grain yield. Correlation showed that there is strong positive correlation between different stage of SPAD with grain yield.

## Introduction

Chlorophyll concentration in the leaves of maize is the most potent factor to trap light energy and utilize the excitation energy to fix atmospheric carbon dioxide [1] into 3-phosphoglycerate, glucose and its derivatives. Leaf chlorophyll is the principal photosynthetic biochemical which contain majority of leaf nitrogen damages the leaf chlorophyll and then subsequently lowers photosynthetic efficiency of maize [2].

Leaf SPAD observations are collinearly correlated with leaf chlorophyll content for several crops [3]. Dwyer et al. [4] stated that central leaves in the maize plants have higher N concentration before anthesis and then starts declining up to 2-week after the anthesis. Transformation equation of Dwyer et al. [4] yields higher N concentration from SPAD value as X in independent variable before anthesis than after 2-week after the anthesis. But the N concentration is highest in the central leaves when the plants cross the age of live weeks alter anthesis in comparison to pre-anthesis to fourth week of anthesis [4]. The SPAD observation obtained from it is highly and positively correlated with leaf Chlorophyll and N contents [5].

Phenotyping using chlorophyll meter Soil Plant Analyzer Development (SPAD)-502 (SPAD-502 Konica Minolta Sensing Inc., Japan) (Minolta Camera Co Ltd, 1989) gives SPAD reading to indicate index of chlorophyll a and chlorophyll b in thylakoid membrane in the leaf mesophyll chloroplasts. Simultaneously, the device has been used to estimate leaf N concentration from the SPAD measure. Dwyer et al*.* [4] displayed that the SPAD measure at central leaves is correlated to the leaf N content in slightly quadratic pattern in maize.

## Main text

### Materials and methods

The experimental materials consist of Five register hybrids of multinational seed companies (Shresta, Ganga Kaveri, Bisco 940 New, P3396 and Rajkumar) three recently released hybrid (Rampur hybrid-2, Rampur hybrid-4 and Rampur hybrid-6) and two pipeline hybrids (RML-86/RML-96 and RML-95/RML-96) developed by National Maize Research Program (NMRP), Rampur, Chitwan were evaluated in RCBD with three replication during 2019 at Rapti-7, Chitwan.

Plots of 9 m^2^ area were made for each genotype received with the net plot area of 90 m^2^ per replication. Seed was sown at the rate of two seeds per hill with the crop geometry of 75 $\times$ 25 cm^2^ (RR $\times$ PP). All inter-culture operations were carried out as per national recommendations. SPAD measurement at vegetative stage was taken from T1 leaves and T3 leaves of five plants from each plot during 30 days interval.

During reproductive phase data were taken from eo leaves, e2 leaves throughout period of grain filling at 10 days intervals. Same leaves were used for overall data collection. Leaf chlorophyll index was measured by using SPAD meter (SPAD 502 plus, Minolta, Japan).

## Results

### SPAD value at vegetative stage and grain filling stage

**Fig. 1:**
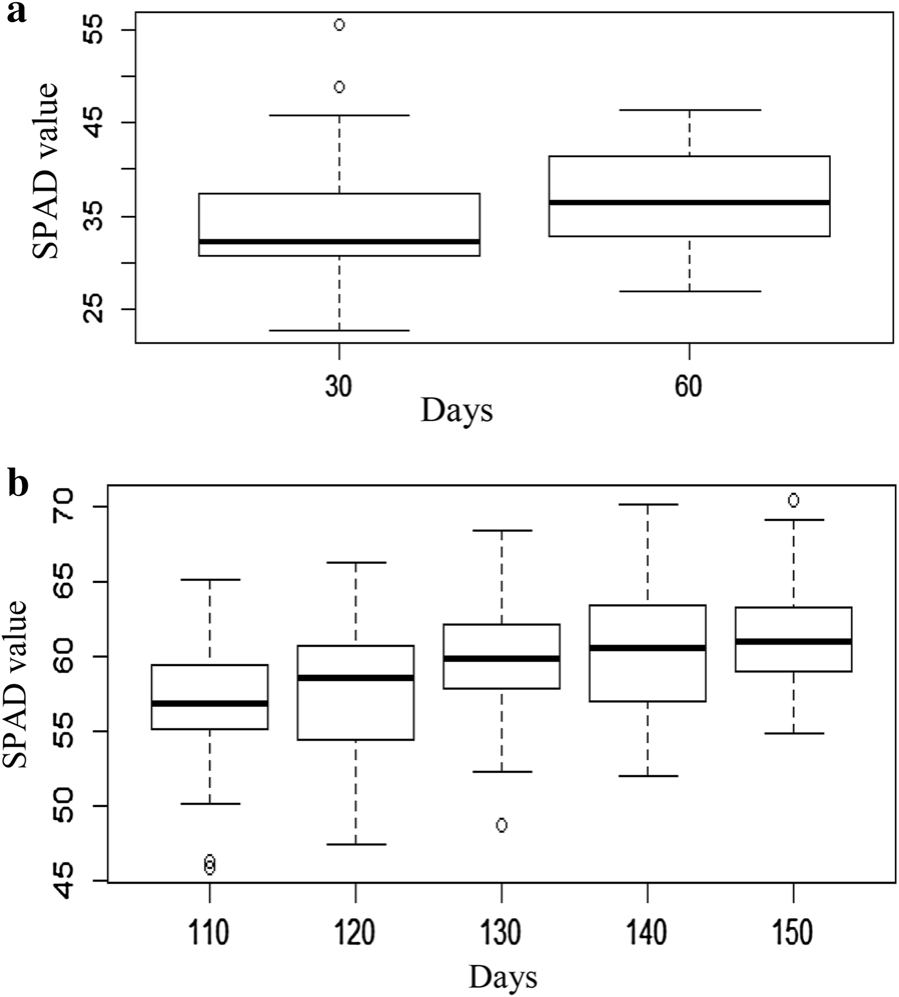
SPAD value varies with different age. **a** At 30 DAS and 60 DAS of vegetative stage. **b** At different period of reproductive stage. The upper and lower limit of each box represent 25th and 75th percentile, o indicate outlier, central  indicates median

There was significant variation in SPAD between 2 different age; 30 DAS and 60 DAS as shown as in Fig. 1a. Higher SPAD value was observed in 60 DAS. Highly significant variation of SPAD value at different age of reproductive stage is observed which is shown in Fig. 1b. SPAD value increased with increased duration of 4–5 weeks, and then its value decreased at the time of harvesting. Some green genotypes showed higher SPAD reading at the time of harvesting.

### SPAD value in maize leaves

**Fig. 2:**
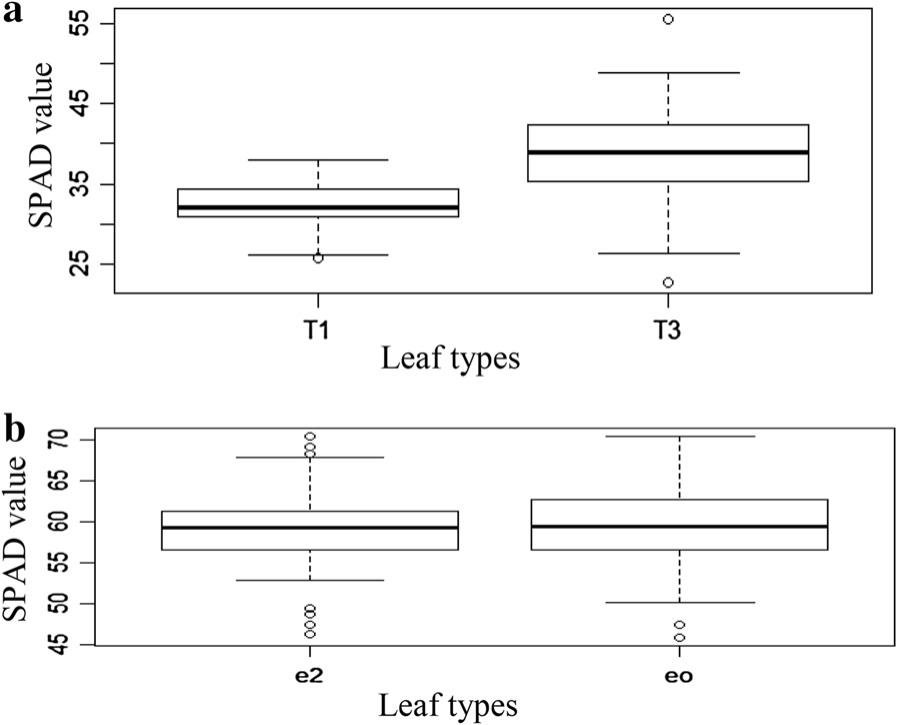
SPAD value varies with different leaf wise. **a** T1 and T3 leaves of vegetative and juvenile stage. **b** eo and e2 of reproductive stage. The upper and lower limit of each box represent 25th and 75th percentile, o indicate outlier, central  indicates median

Figure 2 shows that during vegetative growth stage, we recorded top most leaf with lower SPAD value as compared to 2nd leaf from topmost leaf and effect were significant different during vegetative growth stage. Top most leaves had lower SPAD value because they were not fully open. At grain filling stage significantly different SPAD value was found among 2 leaves. eo leaves had higher SPAD value and more contribution to grain yield than e2 leaves.

### Correlation between different stage SPAD values with grain yield

T1 leaf at 30 DAS was highly significant and positively correlated with grain whereas T3 leaf at 30 days was significantly positive correlation with grain yield shown in Table 1. At 60 DAS both T1 and T3 leaf showed positive highly significant correlation with grain yield. eo and e2 leaf after grain filling stage were found to be highly positive significant association with grain yield shown in Table 1.

**Table 1:** Correlation between different stage and leaf of SPAD value with grain yield

SPAD value at different age	Correlation coefficient (r) with GY (t ha^−1^)	R^2^
T1 SPAD 30 DAS	0. 48 **	0.24
T3 SPAD 30 DAS	0. 45 *	0.21
T1 SPAD 60 DAS	0.68 **	0.46
T3 SPAD 60 DAS	0.65 **	0.42
eo SPAD 110 DAS	0.74 **	0.55
e2 SPAD 110 DAS	0.60 **	0.36
eo SPAD 120 DAS	0.78 **	0.61
e2 SPAD 120 DAS	0.74 **	0.55
eo SPAD 130 DAS	0.93 **	0.86
e2 SPAD 130 DAS	0.95 **	0.90
eo SPAD 140 DAS	0.74 **	0.55
e2 SPAD 140 DAS	0.72 **	0.52

^**^ Correlation is significant at the 0.01 level (2-tailed), *Correlation is significant at the 0.05 level (2-tailed), T1, top most leaf at early growth stage; T3, 2nd leaf from T1; eo, leaf attach with cob, e2, 2nd leaf from eo leaf; DAS, days after sowing; SPAD, soil plant analyzer development

## Discussions

Leaves at the middle strata of the maize canopy contribute more photosynthates to grain than do other leaves [6], however, leaves at the lower strata may suffer from weak light condition and increase the consumption of respiration which was related to leaf age [7], therefore, the highest yield in the leaf removal treatment (D3 or D4) maybe due to the removal of the lower strata leaves after silking. The middle leaves are the main functional leaves for dry-matter production, and they obtain more solar radiation when the leaves in the upper canopy are upright [8]. Adhikari et al. [9] evaluate fifteen newly bred single cross hybrids of yellow maize in term of chlorophyll and N concentration on e_0_ and e_3_ leaves of maize hybrids in winter reported that e_0_ leaf has been found more grain yield determining than e_3_ leaf.

The high yield potential hybrids might have highly efficient photosynthetic apparatus on e_0_ than the e_3_ leaf; and efficient leaf nutrient mobilization efficiency from protein degradation to the kernels during crop maturity [10]. Besides, it can also be said that non-collinear correlation between the SPAD and grain yield can also reflect differential strength in N pulling, chlorophyll synthesis, chlorophyll and soluble protein degradation among different leaves and different genetic system of the hybrids.

Ghimire et al. [11] reported that positive and significant effect of chlorophyll content in grain yield of the maize. In many studies, leaf chlorophyll value measured by SPAD chlorophyll meter was found closely related to grain yield [12–14] which were all in accordance to our findings.

## Conclusion

Overall, results from our research suggest that SPAD value increase with age and decline at the time of harvesting. Leaves eo contribute more to grain yield. SPAD value of different age and leaf are positively associated with grain yield.

## Limitation of the study

This limitation to this study mainly focuses on SPAD of two leaves (i.e. eo leaf and e2 leaf at reproductive stage) only not coverage up to top leaves (i.e. e4, e6 and so on). Similarly, its only coverage T1 and T3 leaves for study not all leaves upon consideration.

## Data Availability

The raw data used in this research cannot be shared at this time as the data also form parts of an ongoing study, but data are available from corresponding authors upon reasonable request.

## Abbreviations

SPAD: Soil Plant Analyzer Development
eo: Leaf attach to cob/ear
e2: 2nd leaf from eo leaf
T1: Top most leaf at vegetative and juvenile satge
T3: 2nd leaf from T1 leaf
N: Nitrogen
GY: Grain yield
DAS: Days after sowing
